# Trends in the uptake of nipple sparing mastectomy in *BRCA1/2*,* PALB2* and other pathogenic variant carriers undergoing risk reducing mastectomy

**DOI:** 10.1186/s13053-026-00351-0

**Published:** 2026-07-24

**Authors:** Marya Alsuhaibani, Amel Melanson, Alex Viezel-Mathieu, Tassos Dionisopoulos, Mark Basik, Jean Francois Boileau, Karyne Martel, Sarkis Meterissian, Joshua Vorstenbosch, Victor Villareal-Corpuz, William D. Foulkes, Ipshita Prakash, Stephanie M. Wong

**Affiliations:** 1 Department of Surgery, McGill University, Montreal, QC Canada; 2 Stroll Cancer Prevention Centre, Jewish General Hospital, Montreal, QC Canada; 3 Department of Plastic and Reconstructive Surgery, McGill University, Montreal, QC Canada; 4 Department of Oncology, McGill University, Montreal, QC Canada; 5 Lady Davis Institute for Medical Research, Jewish General Hospital, Montreal, QC Canada; 6 Department of Human Genetics, McGill University, Montreal, QC Canada; 7 Segal Cancer Centre, Jewish General Hospital, 3755 Cote Ste Catherine, E713, Montreal, QC H3T1E2 Canada

**Keywords:** *BRCA1/2*, Hereditary breast cancer, Nipple sparing mastectomy, Skin sparing mastectomy, Risk reducing mastectomy

## Abstract

**Introduction:**

Risk-reducing mastectomy (RRM) for germline pathogenic variant (GPVs) carriers were historically performed as total mastectomy (TM) or skin-sparing mastectomy (SSM), although nipple-sparing mastectomy (NSM) has recently emerged as an oncologically safe RRM option.

**Methods:**

This retrospective cohort study included all female patients with confirmed *BRCA1*,* BRCA2*,* PALB2*, or other high-penetrance GPVs at two academic institutions in Canada between 2003 and 2024. Temporal trends in NSM uptake were assessed using the Mantel-Haenszel test for trend and multivariable logistic regression.

**Results:**

After exclusions, 559 GPV carriers including 319 (57%) affected carriers and 240 (43%) unaffected carriers comprised the study cohort. The median age at RRM was 45 years old (range 22–76) and most were *BRCA1* carriers (46.7%) or *BRCA2* carriers (44.4%). Overall, 310 carriers (55.5%) underwent NSM, 174 (31.1%) underwent SSM, and 75 (13.4%) underwent TM. NSM uptake was highest among patients aged 40–49 years (56%) and lowest in those ≥ 60 years (42%, *p* = 0.03). Relative to unaffected carriers, affected carriers (47% vs. 66%, *p* < 0.001) and those with prior radiation were less likely to undergo NSM (34.3% vs. 62.7%, *p* < 0.001). A clear increase in NSM occurred over time, from 11.9% pre-2012 to 74.4% after 2022 (*p* < 0.001). Multivariable analysis adjusting for age, GPV, parity, menopausal status, and personal history of breast cancer identified normal BMI (OR 1.99, 95% CI 1.18–3.34), lack of radiation exposure (OR 3.03, 95% CI 1.76–5.23), and year of surgery as independent predictors of NSM receipt (OR 1.25, 95% CI 1.19–1.32).

**Conclusion:**

There has been a significant increase in NSM use among *BRCA1/2*,* PALB2* and other GPV carriers over the last two decades.

## Introduction

Germline pathogenic variants (GPVs) in high penetrance breast cancer susceptibility genes such as *PALB2* and *BRCA1/2* confer an estimated 50–70% lifetime risk for developing breast cancer [1, 2]. For individuals carrying these pathogenic variants, risk reducing mastectomy (RRM) is considered the most effective measure to prevent the development of breast cancer. In early studies, RRM was shown to reduce risk by 90–95% [3, 4]. Improvements in reconstructive techniques combined with widespread coverage of RRM in the lay media have increased adoption and uptake of RRM amongst eligible carriers over the last decade [5, 6]. 

In the past, RRM were mainly performed as total mastectomy or skin-sparing mastectomy (SSM). However, over the last decade, nipple-sparing mastectomy (NSM) has increasingly become an appealing and popular surgical option. In NSM, preservation of the nipple-areolar complex results in improvements in aesthetic outcome, quality of life, and patient satisfaction with body image [7–10]. Initially, concerns around the oncologic safety of NSM centred around the possibility of leaving residual glandular tissue behind, which may serve as a focus for future cancer development. However, recent single and multicentred cohort studies have shown acceptably low or negligible rates of subsequent breast cancer in *BRCA1/2* carriers undergoing NSM [8, 11–13]. 

Despite its use in standard practice, there remains a lack of knowledge around the real-world adoption of NSM among pathogenic variant carriers. Understanding these trends are important to improve decision making, standardization of care, and to provide information so that carriers may make informed choices around preventive options. The purpose of this study was to evaluate trends in NSM uptake within a large multicentered cohort of women with pathogenic variants in high penetrance breast cancer susceptibility genes, including *BRCA1*/2, *PALB2* and others.

## Methods

### Cohort selection

This retrospective cohort study includes all female patients with a confirmed pathogenic or likely pathogenic variant in *BRCA1*/2, *PALB2*,* CDH1*,* PTEN*, or *TP53* with a history of RRM seen at the Jewish General Hospital Segal Cancer Centre or McGill University Health Center, in Montreal, Canada, between January 2003 to December 2024. Patients with a variant of uncertain significance or those without confirmed pathogenic variants were excluded. Before commencing the study, research ethics board approvals were obtained from the Jewish General Hospital (CIUSSS West-Central Montreal Research Ethics Board, reference no. MP-05-2024-3780) and McGill University Health Centre (CIUSSS Central West of the Island of Montreal Research Ethics board). Due to the use of retrospective, de-identified data obtained from the medical record, consent from participants was deemed unnecessary. Data was then collected from all paper and electronic medical records and entered into a REDCap database housed at the Jewish General Hospital’s Lady Davis Institute for Medical Research.

### Outcome of interest

The primary outcome of this study was type of RRM, evaluated as the proportion of pathogenic variant carriers undergoing NSM compared to SSM or total (simple) mastectomy over time. Risk reducing mastectomy was defined as any unilateral (contralateral) or bilateral RRM performed for the purposes of breast cancer prevention. Patients undergoing subcutaneous mastectomy, typically used in the early 2000s and often considered a ‘first-generation’ NSM, were placed within the NSM subgroup. Nipple sparing mastectomies were typically performed via radial, circumareolar, or lateral inframammary incisions at the discretion of the surgical team. Patients undergoing wise pattern reduction type incisions with preservation of the nipple-areola complex on a de-epithelialized inferior dermal pedicle were also placed within the NSM group. Nearly all NSM and SSM patients underwent reconstruction, whereas total mastectomy patients underwent no reconstruction or delayed reconstruction at a later time point.

### Independent variable of interest

Independent variables collected included age in years at the time of RRM, germline pathogenic variant (*BRCA1*, *BRCA2*, *PALB2* or other), parity, menopausal status, BMI at surgery (< 18.5, 18.5–24.9, 25-29.9, or 30+), personal history of breast cancer (affected vs. unaffected carrier), smoking status (never, former, or current smoker), medical comorbidities (including diabetes), and prior receipt of radiation (yes vs. no). Breast surgery related information included date of surgery, presence of reconstruction (yes vs. no), type of reconstruction (direct to implant, autologous, or tissue expander) and for implant-based appraoches, implant placement (pre-pectoral, retropectoral, dual plane).

### Statistical analysis

Descriptive statistics and univariate analyses were performed using the Wilcoxon rank sum test for continuous variables and Chi-squared testing for categorical variables. To evaluate temporal trends, we calculated the proportion of patients undergoing each surgical approach (total mastectomy without reconstruction, SSM with reconstruction, and NSM with reconstruction) using the Mantel-Haenszel test for trend. An adjusted multivariable model was then used to assess which of the variables of interest were independently associated with increased odds of NSM. Model selection was performed with all significant variables on univariate analysis included in the adjusted model. Early post-operative complications and reoperation rates were compared across groups using the Chi-squared test, with 5-year cumulative incidence of supplemental surgery measured using the cumulative incidence function and compared using Gray’s test. All statistical analyses were performed using the Statistical Analysis System (SAS) software version 9.4 (Cary, NC).

## Results

### Cohort characteristics

**Fig. 1:**
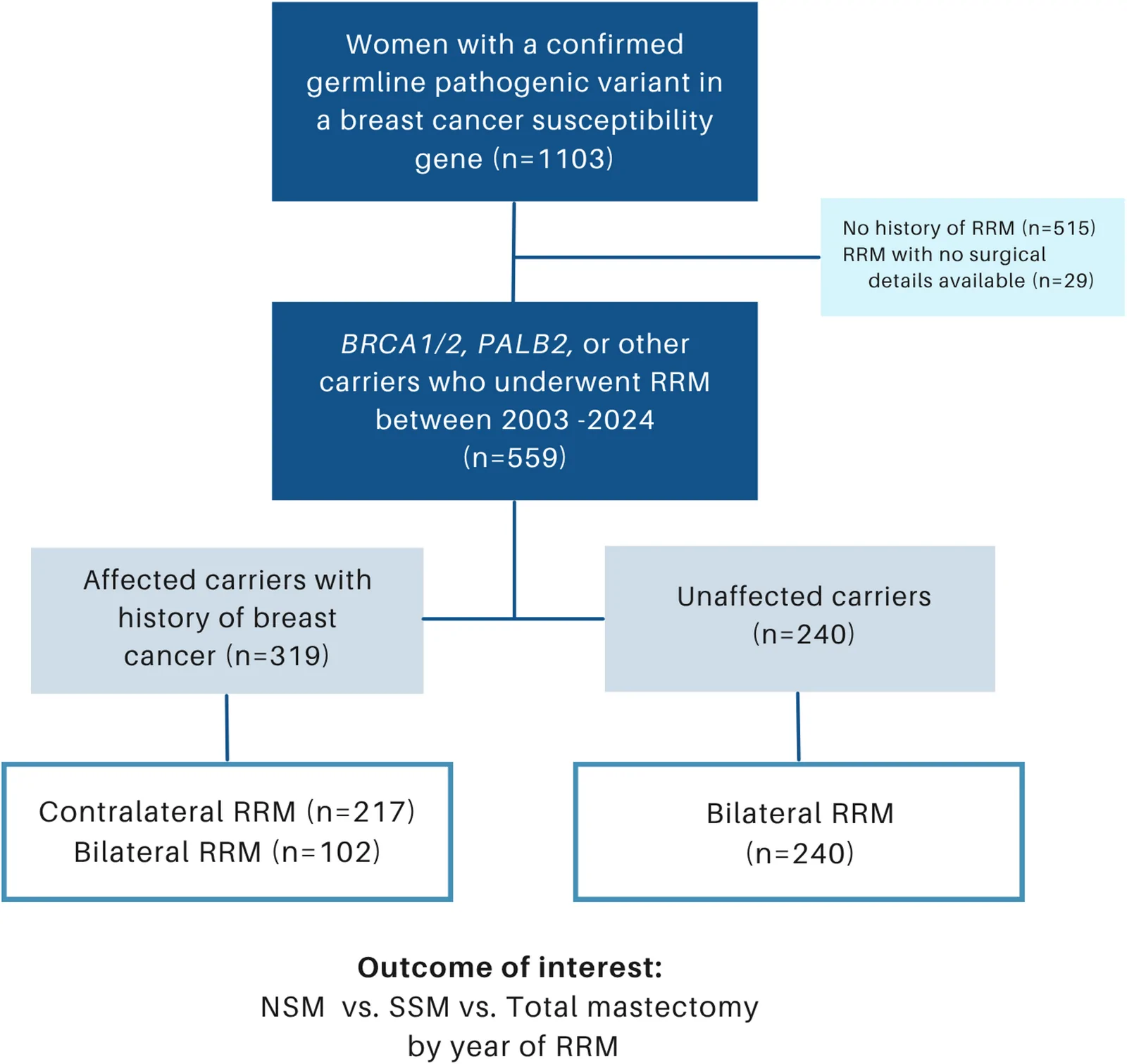
Cohort selection. NSM nipple sparing mastectomy; RRM risk reducing mastectomy. SSM skin sparing mastectomy

Of 1103 pathogenic variant carriers, 588 (53.3%) had germline pathogenic variants in *BRCA1/2*,* PALB2*, or other high penetrance breast cancer susceptibility genes and had undergone RRM. After excluding 29 cases with unknown surgical details, 559 individuals were included in the final analytic cohort (Fig. 1). Among them, 319 (57%) had a personal history of breast cancer and 240 (43%) were unaffected carriers. In the 319 patients with a personal history of breast cancer, 217 (68.0%) women had unilateral therapeutic mastectomy with contralateral RRM, while 102 (32.0%) underwent initial breast-conserving surgery followed by bilateral RRM. The median age at the time of RRM was 45 years (range 22–76), with an older median age at RRM seen in affected carriers compared to unaffected carriers (47 vs. 42 years, *p* < 0.001). Most individuals carried a *BRCA1* (46.7%) or *BRCA2* (44.4%) pathogenic variant, although 50 (8.9%) carried a germline pathogenic variant in *PALB2*,* CDH1*,* PTEN*, or *TP53*. Detailed clinical characteristics are listed in Table 1.

### Temporal trends in risk reducing mastectomy approach

**Fig. 2:**
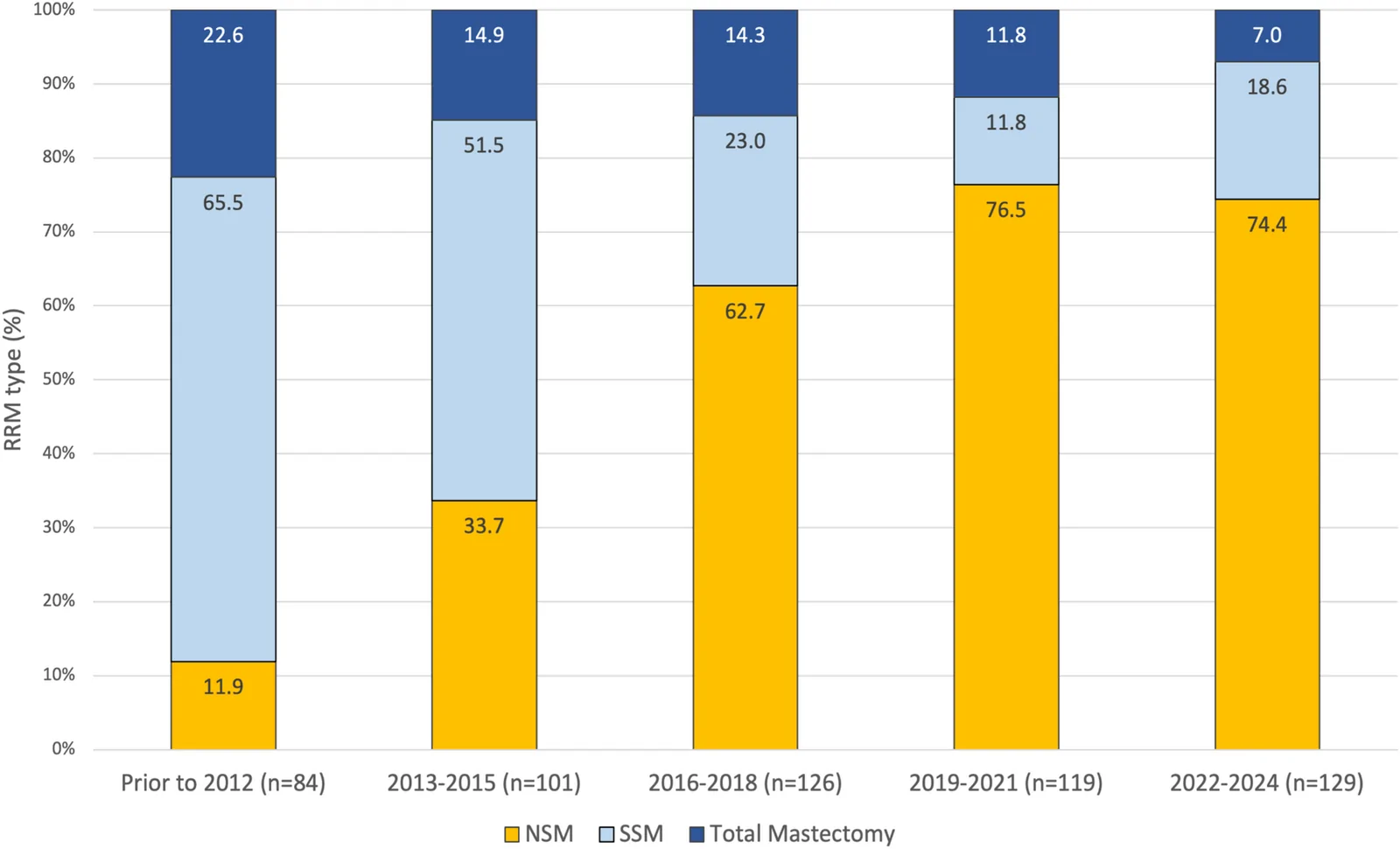
Type of risk-reducing mastectomy performed by year of surgery. SSM was the most common type of RRM performed early in the study period (65.5% before 2012), following which there was a clear trend towards increasing use of NSM, from 12% of cases performed before 2012 to 74% of cases after 2022 (*p* < 0.001). After 2016, it remained the dominant RRM approach for *BRCA1*/2 and *PALB2* carriers

Of the 559 included subjects, 310 (55.5%) underwent NSM, 174 (31.1%) underwent SSM, and 75 (13.4%) underwent total mastectomy without reconstruction. Throughout the study period, 23 different surgeons performed at least one RRM, with the top 5 high-volume surgeons performing 357 (63.8%) of 559 documented RRMs total and 226 (72.9%) of 310 NSMs. The first documented subcutaneous mastectomy was performed in 2003 at the McGill University Health Centre, after which there was negligible use of the procedure until the subsequent adoption of NSMs in 2010 at the McGill University Health Centre, followed by 2013 at the Jewish General Hospital Segal Cancer Centre. Prior to 2012, most women underwent SSM (65.5%) compared to only 18.6% of patients after 2022 (*p* < 0.001, Fig. 2). The use of total mastectomy (without reconstruction) also decreased over the study period, from 22.6% of carriers undergoing RRM prior to 2012 to only 7.0% after 2022. Over the study period, there was a clear trend towards increasing use of NSM in carriers, from 11.9% of cases performed prior to 2012 to 74.4% of cases after 2022 (*p* < 0.001, Fig. 2).

### Factors associated with uptake of nipple sparing mastectomy in BRCA1/2 and PALB2 carriers

Table 2 shows demographic and clinical characteristics associated with NSM. Uptake of NSM varied significantly throughout age groups, with the greatest rates seen among those aged 40–49 years (60.9%) and lowest uptake among those aged 60 years and older (42%, *p* = 0.03). Germline pathogenic variant also affected NSM uptake, with *PALB2*,* TP53*,* CDH1* or *PTEN* carriers having the highest rates (78.0%), compared to *BRCA1* carriers (51%, *p* = 0.02). Unaffected carriers were more likely to undergo NSM compared to affected carriers (66.7% vs. 47%, *p* < 0.001), and patients with prior radiation were significantly less likely to undergo NSM (34.4% vs. 62.7%, *p* < 0.001).

Table 3 displays the odds ratios from a multivariable logistic regression model evaluating predictors of NSM, with adjustment for age group, germline pathogenic variant, parity, menopausal status, BMI, personal history of breast cancer, prior radiation, and year of RRM. In adjusted analyses, BMI at surgery (*p* = 0.02), prior radiation (*p* < 0.001), and year of RRM (*p* < 0.001) remained significantly associated with NSM receipt. The strongest predictor was year of surgery; each additional year following 2003 was associated with a 25% increase in NSM receipt (OR 1.25, 95% CI, 1.19–1.32). Lack of prior radiation also predicted NSM receipt (OR 3.03, 95% CI 1.76–5.22, *p* < 0.001). Compared to those who were underweight (BMI < 18.5) or overweight (BMI 25 or more), those within a normal weight range with a BMI 18.5–24.9 had increased odds of NSM (OR 1.99, 95% CI 1.18–3.34). Other factors such as age, germline pathogenic variant, parity, menopausal status, and prior history of breast cancer did not remain statistically significant in the multivariable model.

### Complications and reoperation rates across risk reducing mastectomy approaches

Early post-operative complications, defined as any infection, hematoma, persistent seroma, loss of the nipple areola complex, flap necrosis, implant exposure and/or prosthesis removal occurring within 30 days of the index RRM were similar between mastectomy approaches, occurring in 38 of 310 (12.3%) NSM patients, 19 of 174 (10.0%) SSM patients, and 6 of 75 (8%) total mastectomy patients (*p* = 0.57). At a median follow up of 5.3 years (IQR, 2.4–8.8 years), supplemental surgeries, defined as any operation requiring general or local anesthesia performed outside of the initially planned procedure(s), occurred in 106 (34.2%) of NSM patients, 55 (31.6%) SSM patients, and 8 (10.7%) of total mastectomy without reconstruction patients, primarily for capsular contracture. The 5-year cumulative incidence of supplemental surgery was 37.6% (95% CI, 31.4–43.8) for NSM, 32.2% (95% CI, 24.7–40.0%) for SSM, and 10.9% (95%CI, 6.0-32.3) for total mastectomy without reconstruction (*p* < 0.001).

## Discussion

In this retrospective cohort study of 559 predominantly *BRCA1*/2 and *PALB2* carriers undergoing 901 RRM over two decades, there was a substantial increase in the proportion of patients undergoing NSM for surgical risk reduction. The use of NSM rose from less than 15% of RRM performed before 2012 to more than 70% of RRM procedures performed after 2022. In multivariable analyses, factors significantly associated with NSM receipt included normal BMI, lack of prior radiation, and more recent year of RRM. These data underscore the gradual and sustained shift in practice patterns towards nipple preservation in high-risk patients since the late 2000s.

In the early 2000s, proponents of NSM commonly cited improved cosmesis, patient satisfaction, and psychosexual well-being as important factors driving uptake [7–9, 12], while others expressed concerns that sparing the nipple would compromise surgical prevention through retention of subareolar terminal ductal lobular units. This concern appeared to be borne out by early reports that showed a slight, non-significant increase in subsequent breast cancer in those treated with ‘subcutaneous mastectomy’, an older NSM approach that left up to 10 mm of subareolar tissue in situ to maintain nipple viability [3]. When ex vivo anatomic studies subsequently demonstrated that it was safe to remove nearly all ductal tissue under the nipple dermis while minimizing ischemic complications [14, 15], NSM began to be more widely adopted for both surgical risk reduction and therapeutic mastectomy purposes [8, 16]. 

Compared to SSM, residual breast glandular tissue is known to be higher following NSM [17], although whether this translates into clinically meaningful differences in long-term breast cancer risk remains unverified. In volumetric MRI analysis of 105 *BRCA1/2* patients, the proportion of total breast glandular tissue present within a 5 mm subareolar depth was only 1.3%, suggesting that keeping the overlying nipple areola complex likely adds minimal oncologic risk [18]. In a large, multicentered cohort study of 548 NSM performed in 346 *BRCA1/2* carriers published in 2017 in the United States, no new breast cancers were diagnosed on the side of the NSM procedure at 34 months follow-up [13]. These data form the best available evidence for the oncologic safety of NSM for risk reduction. In our study, we saw the fastest growth in NSM use in the years following its publication; after 2017, NSM became the predominant surgical approach in the majority of carriers undergoing RRM.

The increased uptake of NSM in pathogenic variant carriers mirrors temporal trends reported in the literature of women with breast cancer [19]. In a large, population-based National Cancer Database (NCDB) study of 157,247 patients undergoing mastectomy and reconstruction for breast cancer, the use of NSM increased from 1.7% in 2005 to 14.3% in 2015 nationwide [16]. In another single institution study from Italy, the use of NSM as a proportion of all mastectomies increased from 21% in 2009 to 67% in 2018 [20]. In our study, the use of NSM increased from 12% prior to 2012 to 63% between 2016 and 2018, suggesting that the widespread adoption and standardization of the technique amongst breast surgeons likely influenced the acceptance of NSM for high-risk patient populations as well [21]. Studies exploring incision placement with NSM suggest that lateral inframammary fold incisions may minimize complications [22] and optimize nipple-areolar complex sensation [23], making it the most popular incision reported in the available literature [24]. 

Our analysis did reveal two additional factors negatively associated with the use of NSM in carriers; radiation exposure and BMI. These risk factors have been widely reported in the literature; relative to non-irradiated patients, prior radiation and PMRT have been shown to increase the risk of complications post-NSM from 10% to 18–22%, respectively [25]. However, increased complications have also been reported in irradiated patients who undergo SSM, where as many as one fifth of cases may develop infection, expander/implant loss, or wound breakdown [26]. While disclosure of these attendant risks are important in pre-surgical discussions, patients should be reassured that implant loss occurs in fewer than 8% of NSM cases where radiation has been administered or is required post-mastectomy [27]. Overall, radiation increases the possibility of complications and revisional surgery, but reconstructive failure remains a relatively rare event.

In our study, patients with low BMI were more likely than those at normal or elevated BMI to elect for no reconstruction, while patients with BMI (> 30) were more likely to be offered skin-sparing approaches for RRM. In the literature there are data to suggest increasing BMI is associated with a higher odds of ischemic complications during NSM, possibly due to larger breast size, greater surface area of dissection, and longer sternal notch-to-nipple distance [28–30]. At our institutions, particularly for carriers undergoing elective RRM who have larger breast size and/or moderate-severe ptosis, a staged approach with initial bilateral reduction mastopexy followed by bilateral RRM with reconstruction is preferred to minimize the risk of ischemic complications.

Concurrent with mastectomy approaches, reconstructive trends have evolved over time. Using a similar cohort of 443 carriers, our group recently demonstrated increased use of reconstruction, from 85% of carriers undergoing RRM prior to 2012 to 97% after 2022 (*p* < 0.01) [31]. We also demonstrated that while implant-based reconstruction was the dominant reconstructive approach - used in over 90% of carriers undergoing RRM - autologous/flap-based techniques steadily increased over time (from 2% of all reconstructive procedures prior to 2012 to 13% after 2022). For patients undergoing implant-based reconstruction, direct-to-implant prepectoral approaches (either single-staged or staged following reduction mastopexy) were used in nearly two thirds of patients after 2022, suggesting a trend away from tissue expander use at our institutions.

Our study included 23 *PALB2* pathogenic variant carriers, who had the highest uptake of NSM on univariate analysis. However, this is likely attributed, in part, to the recent characterization of *PALB2* as a major breast cancer susceptibility gene in 2014 [32], at the same time that NSM was becoming widely adopted. The formal inclusion of *PALB2* as a high penetrance breast cancer susceptibility gene warranting consideration for RRM was limited in guidelines prior to 2018 [33, 34]. Notably, this would mean that *PALB2* carriers among our study population were likely ascertained and counseled about RRM once NSM had become established as standard of care at the involved institutions.

Results of this research should be viewed in light of certain limitations. Firstly, the retrospective nature of this study increases the risk of selection or information bias. Given the lack of prospectively collected data, we cannot be certain that included cases reflect all consecutive RRMs performed at the respective institutions, as missing surgical details resulted in the exclusion of a subset of patients. Secondly, trends may reflect practice in academic, comprehensive cancer centers with high breast surgical volumes but may not be generalizable to all centers (including community hospitals) performing breast surgery, and data on surgical decision making (particularly if NSM was offered or not discussed) are lacking. Finally, follow-up of patients was not consistent across institutions, making it difficult to evaluate long-term complications or cancer outcomes. Despite the stated limitations, the results of this research and its long-time span, as well as its inclusion of over 550 *BRCA1/2*,* PALB2*, and other high penetrance carriers, provides convincing evidence of evolving real-world surgical practices for RRM, with NSM becoming the standard of care over the last decade.

## Conclusion

Our study demonstrates a dramatic increase in NSM uptake among germline pathogenic variant carriers over the past two decades, possibly driven primarily by accumulating safety data, increased surgeon comfort and evolving patient-centered priorities. Ongoing long-term follow-up and continued evaluation of oncologic outcomes are essential to further refine risk-reducing strategies and ensure optimal, evidence-based care for high-risk women.

**Table 1:** Cohort characteristics

Characteristic	(***n*** = 559)
**Median age at RRM –** *years***, (range)**	45 (22–76)
**Germline Pathogenic Variant –** ***n*, (%)**	
* BRCA1* * BRCA2* * PALB2* Other	261 (46.7) 248 (44.4) 23 (4.1) 27 (4.8)
**Parity – ** ***n*** **, (%)**	
Nulliparous One or more children	130 (23.3) 429 (76.7)
**Menopausal status –** ***n*, (%)**	
Premenopausal Post-menopausal after premenopausal RRSO Post-menopausal after natural menopause (+/- RRSO)	225 (42.0) 186 (34.7) 125 (23.3)
**BMI at surgery –** ***n*, (%)**	
<18.5 18.5–24.9 25-29.9 30 or more	177 (31.7) 197 (35.2) 102 (18.3) 83 (14.9)
**Smoking –** ***n*, (%)**	
Never smoker Former smoker Current smoker	433 (77.5) 68 (12.2) 58 (10.4)
**Diabetes –** ***n*, (%)**	
Yes No	22 (3.9) 537 (96.1)
**Breast cancer –** ***n***, **(%)**	
Affected carrier Unaffected carrier	319 (57.1) 240 (42.9)
**Prior Radiation –** ***n*, (%)**	
Yes No	143 (25.6) 416 (74.4)
Year of RRM* – ***n*, (%)**	
Prior to 2012 2013–2015 2016–2018 2019–2021 After 2022	84 (15.0) 101 (18.1) 126 (22.5) 119 (21.3) 129 (23.1)

BMI body mass index; RRM risk reducing mastectomy; RRSO risk reducing salpingo-oophorectomy; *Bold if statistically significant, *p* < 0.05

**Table 2:** Factors associated with nipple sparing mastectomy in *BRCA1/2*,* PALB2* and other carriers undergoing risk reducing mastectomy

Characteristic		Cohort		Proportion undergoing NSM		
		No. (%)		%	*p*-value*	
**Age Group**						
<30 years 30–39 years 40–49 years 50–59 years 60 + years		51 (9.1) 137 (24.5) 179 (32.0) 123 (22.0) 69 (12.3)		56.9 60.6 60.9 48.8 42.0	0.03	
**Germline Pathogenic Variant**						
* BRCA1* * BRCA2* * PALB2*,* TP53*,* CDH1* or *PTEN*		261 (46.7) 248 (44.4) 50 (8.9)		51.0 55.7 78.0	**0.002**	
**Parity**						
Nulliparous Parous		130 (23.3) 429 (76.7)		63.9 52.9	**0.028**	
**Menopausal status**						
Premenopausal Post-menopausal after premenopausal RRSO Post-menopausal after natural menopause (+/- RRSO)		225 (42.0) 186 (34.7) 125 (23.3)		64.9 52.7 46.4	**0.005**	
**BMI at surgery**						
<18.5 18.5–24.9 25-29.9 30 or more		177 (31.7) 197 (35.2) 102 (18.3) 83 (14.9)		44.1 66.0 56.9 53.0	**0.004**	
**Smoking**						
Never smoker Former smoker Current smoker		433 (77.5) 68 (12.2) 58 (10.4)		55.0 54.4 60.3	0.72	
**Diabetes**						
Yes No		22 (3.9) 537 (96.1)		40.9 56.1	0.16	
**Breast cancer**						
Affected carrier Unaffected carrier		319 (57.1) 240 (42.9)		47.0 66.7	**< 0.001**	
**Prior Radiation**						
Yes No		143 (25.6) 416 (74.4)		34.3 62.7	**< 0.001**	
**Year of RRM**						
Prior to 2012 2013–2015 2016–2018 2019–2021 After 2022		84 (15.0) 101 (18.1) 126 (22.5) 119 (21.3) 129 (23.1)		11.9 33.7 62.7 76.5 74.4	**< 0.001**	

BMI body mass index; NSM nipple sparing mastectomy; RRM risk reducing mastectomy; RRSO risk reducing salpingo-oophorectomy; *Bold if statistically significant, *p* < 0.05

**Table 3:** Adjusted odds ratios for factors associated with nipple sparing mastectomy in *BRCA1/2*,* PALB2* and other carriers undergoing risk reducing mastectomy

Characteristic	OR (95% CI)*	*p*-value*
**Age Group**		
<30 years 30–39 years 40–49 years 50–59 years 60 + years	Ref 1.09 (0.47–2.49) 1.04 (0.45–2.44) 0.64 (0.24–1.69) 0.45 (0.15–1.35)	0.33
**Germline Pathogenic Variant**		
* BRCA1* * BRCA2* * PALB2*,* TP53*,* CDH1*,* PTEN*	Ref 1.10 (0.71–1.70) 2.64 (1.09–6.38)	0.10
**Parity**		
Nulliparous Parous	Ref 0.89 (0.53–1.48)	0.66
**Menopausal status**		
Premenopausal Post-menopausal after premenopausal RRSO Post-menopausal after natural menopause (± RRSO)	Ref 1.42 (0.58–3.48) 0.97 (0.42–2.25)	0.35
**BMI at surgery**		
<18.5 18.5–24.9 25-29.9 30 or more	Ref **1.99 (1.18–3.34)** 1.51 (0.83–2.75) 0.86 (0.46–1.64)	**0.02**
**Breast cancer**		
Affected carrier Unaffected carrier	Ref 1.30 (0.79–2.12)	0.30
**Prior Radiation**		
Yes No	Ref **3.03 (1.76–5.22)**	**< 0.001**
**Year of RRM**		
Each additional year following 2003	**1.25 (1.19–1.32)**	**< 0.001**

BMI body mass index; RRM risk reducing mastectomy; RRSO risk reducing salpingo-oophorectomy; *Bold if statistically significant, *p* < 0.05

## Data Availability

De-identified data will be available from the corresponding author upon reasonable request and local ethics board approval.
